# Correlation between novel hematological parameters and assisted pregnancy outcome in frozen-thawed embryo transfer patients

**DOI:** 10.3389/fcell.2026.1781939

**Published:** 2026-06-02

**Authors:** Xuefen Cai, Xiaohui Yang, Yiqin Chen, Yuehong Li, Tuxiang Jiang, Yan Sun

**Affiliations:** 1 Center of Reproductive Medicine, Fujian Maternity and Child Health Hospital, College of Clinical Medicine for Obstetrics & Gynecology and Pediatrics, Fujian Medical University, Fuzhou, China; 2 Obstetrics and Gynecology Department of the Fifth Hospital of Zhangzhou, Zhangzhou, China; 3 Medical Research Center, Fujian Maternity and Child Health Hospital, College of Clinical Medicine for Obstetrics & Gynecology and Pediatrics, Fujian Medical University, Fuzhou, China; 4 Key Laboratory of Clinical Laboratory Technology for Precision Medicine (Fujian Medical University), Fujian Province University, Fuzhou, China

**Keywords:** clinical pregnancy outcomes, frozen-thawed cycles, inflammation, insulin resistance, new hematological parameters

## Abstract

**Background:**

The relationship between preimplantation hematological profiles and clinical pregnancy rates following frozen embryo transfer (FET) has not been thoroughly investigated.

**Methods:**

A retrospective study was conducted. Participants were categorized into clinical pregnancy (N = 404) and non-pregnancy (N = 313) groups. Novel hematological indices were evaluated using multivariable logistic regression, restricted cubic splines (RCS), and weighted quantile sum (WQS) regression to explore the relationship between exposure effects and clinical pregnancy outcomes. Least absolute shrinkage and selection operator (LASSO) regression was applied to select key predictors. Furthermore, we quantified absolute risk differences and assessed incremental predictive value [area under the curve (AUC), net reclassification improvement (NRI), integrated discrimination improvement (IDI)], utilizing sensitivity analyses to prevent statistical over-adjustment.

**Results:**

The non-pregnancy group showed higher levels of age, BMI, and inflammatory/insulin resistance markers. Spearman analysis indicated correlations between hormones (AMH, LH, E2, T) and specific novel hematological indices. Logistic regression indicated positive associations between Triglyceride glucose index (TyG Index), Triglyceride glucose-body mass index (TyG-BMI), and non-pregnancy. RCS analysis showed a nonlinear relationship for the TyG Index (P for overall<0.001, P for nonlinear = 0.031) and a linear relationship for TyG-BMI (P for overall<0.001, P for nonlinear = 0.174). Across TyG-BMI quartiles, the marginal predicted probability of clinical pregnancy was 63.1% in Q1 and 46.6% in Q4 (an absolute difference of 16.5%). The fully adjusted TyG-BMI model yielded an AUC of 0.677. Incorporating TyG-BMI into the baseline clinical model showed incremental predictive value in this dataset (NRI = 0.264, IDI = 0.020). In sensitivity analyses excluding baseline BMI, the association between TyG-BMI and non-pregnancy remained statistically significant. Finally, WQS regression assigned the highest weight to the TyG Index among insulin resistance indicators, and LASSO regression identified NPAR and TyG-BMI are associated with clinical pregnancy outcomes.

**Conclusions:**

The TyG Index and TyG-BMI are associated with clinical pregnancy outcomes of FET. Evaluating these preimplantation indices may help identify women at higher risk of non-pregnancy.

## 1 Introduction

Infertility represents a state of diminished fertility. Recent years have witnessed a persistent global rise in infertility prevalence, posing significant challenges to sustainable population development (Carson and Kallen, 2021). Assisted Reproductive Technology (ART) has emerged as an effective solution for infertility management. As a crucial component of ART, Frozen-Thawed Embryo Transfer (FET) not only safeguards embryos from *in vitro* fertilization (IVF) or intracytoplasmic sperm injection (ICSI) cycles but also significantly enhances cumulative pregnancy and live birth rates per oocyte retrieval while reducing embryo wastage. Furthermore, FET provides a viable alternative for patients unsuitable for fresh embryo transfer. Despite remarkable advancements in ART techniques - including notable improvements in oocyte yield, fertilization rates, and cleavage rates - clinical pregnancy rates remain stagnant at approximately 50%. Therefore, how to further increase the clinical pregnancy rate has always been a key issue that experts in reproductive medicine are concerned about.

Growing evidence suggests that underlying health conditions, such as immune dysregulation from chronic low-grade inflammation and metabolic disturbances induced by insulin resistance, may be negatively associated with ART outcomes (Rudnicka et al., 2021; Rostamtabar et al., 2021; Moin et al., 2021; Dull et al., 2019; Tortorella et al., 2014; Silva et al., 2020; Yang et al., 2020; Niu et al., 2014; Azkargorta et al., 2018; Vexø et al., 2023; Bellver et al., 2021; Li et al., 2024; Luo et al., 2023). However, the multifactorial nature of ART outcomes and the diversity of available diagnostic methods present challenges. Comprehensive screening often proves impractical due to temporal and financial constraints. Therefore, a thorough analysis of routine infertility screening results becomes imperative to evaluate maternal readiness for embryo transfer, thereby informing strategies to optimize clinical outcomes. Inflammation-related parameters derived from complete blood cell count represent a series of indicators reflecting the body’s inflammatory status through ratios of blood cell counts. These include the hemoglobin, albumin, lymphocyte, and platelet score (HALP), neutrophil-to-lymphocyte ratio (NLR), platelet-to-lymphocyte ratio (PLR), lymphocyte-to-monocyte ratio (LMR), systemic immune-inflammation index (SII), platelet-to-albumin ratio (PAR), pan-immune inflammation value (PIV), uric acid to high-density lipoprotein cholesterol ratio (UHR), neutrophil proportion to albumin ratio (NPAR), derived neutrophil-to-lymphocyte ratio (dNLR), monocyte-to-lymphocyte ratio (MLR), and neutrophil-monocyte-to-lymphocyte ratio (NMLR). These novel hematological parameters hold significant value in assessing nutritional and immune status, along with the degree of systemic inflammation1. They serve as potential prognostic indicators across various clinical conditions (Farag et al., 2023; Yilmaz et al., 2016; Buonacera et al., 2022; Islam et al., 2024; Zhang et al., 2023; Watanabe et al., 2023; Song et al., 2023; Xu et al., 2023; Shen et al., 2024). Furthermore, insulin resistance-related indices, such as the triglyceride glucose index (TyG Index), triglyceride glucose-body mass index (TyG-BMI), homeostasis model Assessment of insulin resistance (HOMA-IR), homeostasis model assessment of beta cell function (HOMA-β), and metabolic score for insulin resistance (METS-IR), calculated from fasting glucose, triglycerides, and insulin levels, provide sensitive and specific measures of metabolic dysfunction (Nabipoorashrafi et al., 2022; Simental-Mendía et al., 2008). However, the diagnostic validity and specificity of these markers require further clinical validation, particularly in the context of assisted reproductive outcomes, where research remains scarce.

Therefore, this retrospective observational study aims to investigate the correlations between these novel hematological parameters and clinical pregnancy outcomes following FET, with a focus on exploring the potential associations of pregravid immune dysregulation and metabolic dysfunction with assisted reproductive success. Rather than establishing causality, our findings are intended to serve as a hypothesis-generating foundation, providing new insights into potential correlative markers that might inform future prospective strategies for optimizing clinical outcomes in ART patients.

## 2 Materials and methods

### 2.1 Study design and participants

This retrospective observational study included infertile women undergoing their first hormone replacement therapy (HRT)-prepared FET cycle at the Center of Reproductive Medicine at Fujian Maternity and Child Health Hospital between 1 July 2021, and 31 August 2023. The study protocol was approved by the Institutional Review Board of Fujian Maternity and Child Health Hospital (Approval No. 2025KY032). The inclusion and exclusion criteria for the study can be found as follows: (i) Inclusion criteria: (1) Women aged between 20 and 45 years. (2) Infertile women who underwent IVF/ICSI cycles but did not undergo fresh embryo transfer for various reasons and were planning to undergo FET for the first time. (3) Endometrial preparation using HRT protocol in the current FET cycle. (ii) Exclusion criteria: (1) Previous fresh embryo transfer or history of recurrent implantation failure (RIF) after multiple oocyte retrieval cycles. (2) Use of preimplantation genetic screening (PGS)/preimplantation genetic diagnosis (PGD) for embryo selection in the first treatment cycle. (3) Occurrence of ectopic pregnancy following the current embryo transfer. (4) Patients diagnosed with unexplained infertility. (5) Incomplete or missing essential follow-up data. Excluding fresh cycles and previous failed transfers were implemented to eliminate the acute metabolic disruptions of controlled ovarian hyperstimulation (COH) and minimize profound etiological heterogeneity, thereby allowing us to accurately isolate the independent association between the baseline immunometabolic dysregulation and clinical pregnancy outcomes.

To prevent intra-cluster correlation bias arising from repeated measures, the analysis was conducted on a per-woman basis, strictly restricting the inclusion to the first FET cycle for each patient during the study period. The primary outcome was clinical pregnancy, defined as the presence of at least one intrauterine gestational sac detected by transvaginal ultrasound 4–5 weeks after embryo transfer. Consequently, multiple pregnancies (e.g., twin gestations) were included and classified as a positive clinical pregnancy event. Biochemical pregnancies (transient β-hCG elevation without a visible gestational sac) and true negative β-hCG cases were initially combined into the ‘non-pregnancy’ group for the primary analysis.

### 2.2 Endometrial preparation

Patients initially received oral estradiol (Progynova, Bayer, Germany) at 3–9 mg/day for endometrial preparation, starting on cycle days 2–5. The initial dose was determined based on the patient’s prior endometrial response. Transvaginal ultrasound was performed approximately 7 days later, and the dose was adjusted according to endometrial thickness. When endometrial thickness reached ≥8 mm, patients received either intramuscular progesterone (40 mg daily; Progesterone Injection, Zhejiang Xianju, China) or vaginal progesterone gel (90 mg once daily; Crinone, Merck Serono, UK), combined with oral dydrogesterone (10 mg twice daily; Duphaston, Abbott, Netherlands). Day 5 or 6 blastocysts were transferred 5 days after progesterone initiation. After the transfer, the use of estradiol (according to the usage during endometrial preparation) and 10 mg of dydrogesterone (Duphaston, Abbott, Netherlands) twice times daily was continued. And natural vaginal progesterone (Crinone vaginal gel, Merck Serono, UK) at a dose of 90 mg per day was added for 14 days until the β-human chorionic gonadotropin (β-hCG) test was performed.

### 2.3 Clinical outcome assessment

Non-pregnancy was defined as either serum β-hCG <5 mIU/mL at 14 days post-transfer or biochemical pregnancy (initial β-hCG >5 mIU/mL with subsequent suboptimal rise or decline, and no gestational sac identified on serial ultrasound examinations). Clinical pregnancy was confirmed when a positive β-hCG test was followed by transvaginal ultrasound detection of at least one intrauterine gestational sac at 2 weeks post-positive test.

### 2.4 Observational parameters

The most recent pre-embryo transfer hematological parameters were retrieved from the electronic medical records system of the Reproductive Medicine Center at Fujian Maternity and Child Health Hospital, inlcuding: white blood cell count (WBC), neutrophil percentage (NE%), absolute neutrophil count (ANC), absolute lymphocyte count (ALC), absolute monocyte count (AMC), hemoglobin (Hb), and platelet count (PLT), creatinine, uric acid, albumin, fasting glucose, triglycerides, total cholesterol, high-density lipoprotein cholesterol (HDL-C), and fasting insulin. All peripheral blood samples were collected during the actual FET cycle, specifically on the second to third day of the menstrual cycle before endometrial preparation. To minimize the potential confounding effects of diurnal variations and dietary intake, we have explicitly stated that all hematological parameters were measured following an overnight fast of at least 8 h. Furthermore, all blood drawings were strictly performed in the morning between 07:30 and 10:30 a.m. using standardized laboratory protocols.

The clinical characteristics were also collected, which included female age at oocyte retrieval, height, weight, duration of infertility, baseline anti-Müllerian hormone (AMH) level, fertilization method (IVF/ICSI), endometrial thickness on embryo transfer day, and final clinical pregnancy outcome.

The calculated indices derived from the collected parameters were summarized in Supplementary Table S1.

### 2.5 Statistical analysis

All statistical analyses were performed using R (v4.4.2) and Stata. Continuous variables were expressed as mean ± SD or median [*P*_25_, *P*_75_] and compared using Student´s *t*-tests or Mann-Whitney U tests based on normality. Categorical variables were presented as frequencies (%) and analyzed via Chi-square tests. Spearman’s correlation assessed relationships among continuous metabolic and inflammatory markers. A post-hoc power analysis (N = 717, α = 0.05) confirmed the cohort was adequately powered (93.7% and 99.8%) to detect the primary outcome stratified by median TyG Index and TyG-BMI.

For multivariable logistic regression, continuous exposures were categorized into quartiles (Q1 reference). Covariate selection was guided by clinical relevance and a Directed Acyclic Graph (DAG, Supplementary Figure S3). We employed a sequential modeling strategy: Model 1 adjusted for age and baseline BMI; Model 2 further adjusted for infertility duration, fertilization method, treatment cycles, embryo stage/number, basal hormones (AMH, FSH, LH, PRL, E2, T, P), and comorbidities (PCOS, diminished ovarian reserve, endometriosis, genital inflammation). Age, embryo/treatment numbers, infertility duration, and hormones were modeled continuously, while others were categorical.

Advanced modeling included Least Absolute Shrinkage and Selection Operator (LASSO) regression to handle multicollinearity and select robust features (using minimum cross-validated error, λ.min = 0.0347; Supplementary Table S2). Restricted Cubic Splines (RCS) with three knots (fifth, 50th, 95th percentiles) explored non-linear dose-response relationships, setting the overall median as the reference (OR = 1.0) with Model 2 adjustments. Weighted Quantile Sum (WQS) regression (q = 4, 1,000 bootstraps) evaluated combined mixture effects using a positive directional constraint and a 40%/60% training-validation split.

Clinical magnitude was evaluated using marginal predicted probabilities and absolute risk differences (ARD) across TyG-BMI quartiles. Model discrimination and calibration were assessed via the area under the curve (AUC) and the Hosmer-Lemeshow test. Incremental predictive utility was determined by calculating ΔAUC (DeLong’s test), continuous Net Reclassification Improvement (NRI), and Integrated Discrimination Improvement (IDI) after adding novel indices to a baseline clinical model.

Finally, sensitivity analyses were conducted to confirm TyG-BMI robustness: (1) BMI was excluded from all regression models and baseline clinical evaluations to prevent mathematical coupling and over-adjustment; (2) LASSO-selected predictors were forced into the multivariable models to verify TyG-BMI stability. (3) Biochemical pregnancy and BMI were explicitly excluded from regression models.

## 3 Results

### 3.1 Clinical characteristics of the study participants

There were 717 patients included in this study. Based on clinical pregnancy outcomes, the subjects were categorized into two groups: the clinical pregnancy group (56.3%) and the non-pregnancy group (43.7%). Table 1 shows participants’ characteristics. Embryo transfer characteristics and downstream reproductive outcomes stratified by clinical pregnancy status were also summarized in the Supplementary Table S2. Notably, the number and developmental stage of transferred embryos differed significantly between the two groups (*P* < 0.001). Furthermore, downstream reproductive outcomes, including live birth, miscarriage, and ectopic pregnancy, were exclusively assessed and reported within the clinical pregnancy cohort.

**TABLE 1:** Comparison of general data between the two groups.

Variables	Clinical pregnancy group (N = 404)	Non-pregnancy group (N = 313)	Effect size (95% *CI*)	*P*
Age (years), median [IQR]	31.00 (28.00, 33.00)	32.00 (29.00, 35.00)	**−1.000 (-2.000, -1.000)**	**<0.001**
**BMI, n (%)#**	​	​	-	**0.044**
<18.5 kg/m^2^	53 (13.1)	28 (8.9)	​	​
18.5–24 kg/m^2^	274 (67.8)	205 (65.5)	​	​
≥24 kg/m^2^	77 (19.1)	80 (25.6)	​	​
**Type of infertility, n (%)#**	​	​	​	0.122
Primary infertility	221 (54.7)	153 (48.9)	​	​
Secondary infertility	183 (45.3)	160 (51.1)	​	​
Duration of infertility (years, median [IQR])	3.00 (2.00, 4.00)	3.00 (2.00, 4.00)	<0.001 (−2.769 × 10^−5^, 3.973 × 10^−5^)	0.987
Number of treatments, median [IQR]	1.00 (1.00, 2.00)	1.00 (1.00, 2.00)	<0.001 (−4.755 × 10^−5^, 4.828 × 10^−5^)	0.816
Endometrial thickness (mm, median [IQR])	9.20 (8.60, 10.05)	9.20 (8.50, 10.00)	0.100 (−0.100, 0.200)	0.446
**Fertilization method, n (%)#**	​	​	​	0.064
IVF	317 (78.5)	226 (72.2)	​	​
ICSI	87 (21.5)	87 (27.8)	​	​
FSH (mIU/mL, median [IQR])	5.72 (4.75, 6.71)	5.72 (4.68, 6.97)	−0.060 (−0.290, 0.170)	0.604
LH (mIU/mL, median [IQR])	3.78 (2.71, 5.10)	3.40 (2.40, 4.70)	**0.380 (0.100, 0.600)**	**0.003**
PRL (ng/mL, median [IQR])	13.65 (9.25, 20.05)	13.90 (10.00, 19.00)	<0.001 (−0.900, 1.000)	0.960
E_2_ (pg/mL, median [IQR])	29.00 (22.00, 40.00)	28.00 (20.00, 38.00)	2.000 (0.000, 4.000)	0.099
P (ng/mL, median [IQR])	0.24 (0.10, 0.35)	0.20 (0.13, 0.30)	<0.001 (0.000, 0.020)	0.223
T (ng/mL, median [IQR])	0.32 (0.25, 0.40)	0.30 (0.25, 0.38)	0.010 (0.000, 0.030)	0.192
AMH (ng/mL, median [IQR])	5.29 (3.11, 8.21)	3.91 (1.63, 6.71)	**1.330 (0.810, 1.870)**	**<0.001**
**Polycystic ovary syndrome, n (%)#**	​	​	**−12.7% (-21.1% to -4.2%)**	**0.004**
Yes	121 (30.0)	63 (20.1)	​	​
No	283 (70.0)	250 (79.9)	​	​
**Diminished ovarian reserve, n (%)#**	​	​	**30.5% (19.6% to 41.5%)**	**<0.001**
Yes	26 (6.4)	62 (19.8)	​	​
No	378 (93.6)	251 (80.2)	​	​
**Endometriosis, n (%)#**	​	​	3.9% (−13.8%–21.6%)	0.635
Yes	20 (5.0%)	18 (5.8)	​	​
No	384 (95.0)	295 (94.2)	​	​
**Inflammation of the** **Reproductive system, n (%)#**	​	​	−4.7% (−17.1%–7.7%)	0.437
Yes	46 (11.4)	30 (9.6)	​	​
No	358 (88.6)	283 (90.4)	​	​

Data are presented as median [IQR] or n (%), as specified after each variable. # indicate that the *P*-value was calculated using the Chi-square test. For other variables, *P*-values were calculated using the Mann-Whitney U test. The bold values indicate P<0.05.

### 3.2 Comparison of routine hematological parameters

The non-pregnancy group showed elevated WBC, NE%, ANC, and triglycerides but lower HDL-C levels (*P* < 0.05) versus the clinical pregnancy group (Table 2).

**TABLE 2:** Comparison of routine hematological parameters between two groups.

Routine hematological parameters	Clinical pregnancy group (N = 404)	Non-pregnancy group (N = 313)	Effect size (95% *CI*)	*P*
WBC (10^9^/L, median [IQR])	5.96 (5.01, 7.11)	6.32 (5.34, 7.18)	**−0.270 (-0.470, -0.050)**	**0.014**
NE (%, median [IQR])	56.10 (50.05, 61.50)	58.10 (52.70, 63.30)	**−1.900 (-3.100, -0.600)**	**0.004**
ANC (10^9^/L, median [IQR])	3.37 (2.68, 4.16)	3.59 (2.93, 4.29)	**−0.190 (-0.350, -0.040)**	**0.014**
ALC (10^9^/L, median [IQR])	2.02 (1.69, 2.39)	2.01 (1.73, 2.37)	<0.001 (−0.080, 0.070)	0.957
AMC (10^9^/L, median [IQR])	0.38 (0.31, 0.45)	0.38 (0.32, 0.44)	<0.001 (−0.020, 0.010)	0.756
Hb (g/L, median [IQR])	134.00 (129.00, 138.00)	135.00 (129.00, 141.00)	−1.000 (−2.000, 0.000)	0.163
PLT (10^9^/L, median [IQR])	259.50 (218.00, 95.50)	259.00 (223.00, 293.00)	<0.001 (−8.000, 8.000)	0.989
Uric acid (μmol/L, median [IQR])	307.65 (265.10, 345.75)	309.20 (265.10, 363.70)	−3.800 (−13.500, 5.400)	0.412
Albumin (g/L, median [IQR])	45.95 (44.20, 47.40)	46.00 (44.30, 47.40)	<0.001 (−0.300, 0.400)	0.878
HDL-C (mmol/L, median [IQR])	1.45 (1.22, 1.65)	1.38 (1.19, 1.61)	**0.050 (0.000, 0.100)**	**0.031**
Triglycerides (mg/dL, median [IQR])	0.94 (0.79, 1.25)	1.08 (0.8, 1.50)	**−0.120 (-0.180, -0.060)**	**<0.001**
Fasting glucose^#^ (mmol/L, &#8254; x ± s)	5.23 ± 0.39	5.29 ± 0.41	−0.070 (−0.130, −0.010)	0.050
Fasting insulin (μU/mL, median [IQR])	7.40 (6.30, 9.80)	7.80 (6.40, 10.00)	−0.300 (−0.700, 0.020)	0.128

Data are presented as median [IQR] or ‾x ± s, as specified after each variable. Data marked with.

^#^ were normally distributed and analyzed by an independent samples t-test, and the other variables, *P*-values were calculated using the Mann-Whitney U test. The bold values indicate P<0.05.

### 3.3 Comparison of novel hematological parameters

The non-pregnancy group exhibited significantly higher levels of NLR, UHR, NPAR, NMLR, TyG Index, and TyG-BMI compared to the clinical pregnancy group (*P* < 0.05). These parameters were elevated by 4.82%, 4.58%, 3.25%, 3.76%, 14.29%, and 18.07%, respectively, in the non-pregnancy group (Table 3).

**TABLE 3:** Comparison of novel hematological parameters between the two groups.

Novel hematological parameters	Clinical pregnancy group (N = 404)	Non-pregnancy group (N = 313)	Effect size (95% *CI*)	*P*
HALP, median [IQR]	48.02 (38.96, 59.18)	49.10 (39.75, 59.14)	−0.222 (−2.351, 1.977)	0.849
NLR, median [IQR]	1.66 (1.28, 2.14)	1.74 (1.39, 2.19)	**−0.098 (-0.189, -0.008)**	**0.033**
LMR, median [IQR]	5.34 (4.31, 6.50)	5.24 (4.29, 6.49)	0.057 (−0.185, 0.303)	0.631
PLR, median [IQR]	125.78 (102.79, 154.74)	125.54 (106.25, 151.19)	−0.487 (−5.748, 4.943)	0.861
SII, median [IQR]	420.27 (308.06, 567.27)	441.73 (347.72, 587.44)	−24.036 (−51.904, 3.117)	0.083
PAR, median [IQR]	5.72 (4.78, 6.48)	5.61 (4.86, 6.43)	0.017 (−0.172, 0.198)	0.861
PIV, median [IQR]	164.69 (110.82, 240.34)	168.77 (114.35, 249.95)	−8.343 (−21.729, 4.904)	0.218
UHR, median [IQR]	215.10 (170.70, 259.33)	224.96 (176.65, 282.30)	**−11.174 (-21.978, -0.183)**	**0.046**
NPAR^#^, ‾x ± s	1.23 ± 0.18	1.27 ± 0.19	**−0.042 (-0.071, -0.015)**	**0.003**
dNLR, median [IQR]	0.86 (0.83, 0.89)	0.87 (0.84, 0.89)	−0.003 (−0.010, 0.004)	0.376
MLR, median [IQR]	0.19 (0.15, 0.23)	0.19 (0.15, 0.23)	−0.002 (−0.011, 0.007)	0.631
NMLR, median [IQR]	1.86 (1.46, 2.34)	1.93 (1.58, 2.39)	**−0.098 (-0.193, -0.002)**	**0.045**
METS-IR, median [IQR]	8.46 (6.19, 14.08)	9.44 (6.26, 15.97)	−0.697 (−1.567, 0.125)	0.099
TyG index, median [IQR]	0.91 (0.70, 1.18)	1.04 (0.79, 1.38)	**−0.132 (-0.192, -0.074)**	**<0.001**
TyG-BMI, median [IQR]	19.54 (14.40, 25.50)	23.07 (16.59, 30.41)	**−3.559 (-4.973, -2.136)**	**<0.001**
HOMA-IR, median [IQR]	1.79 (1.41, 2.25)	1.84 (1.48, 2.39)	−0.088 (−0.188, 0.009)	0.077
HOMA-β, median [IQR]	89.67 (69.19, 115.48)	90.14 (70.59, 117.65)	−0.931 (−6.160, 4.200)	0.737

The bold values indicate P<0.05.

### 3.4 Correlation analysis between basal hormones and novel hematological parameters

**FIGURE 1:**
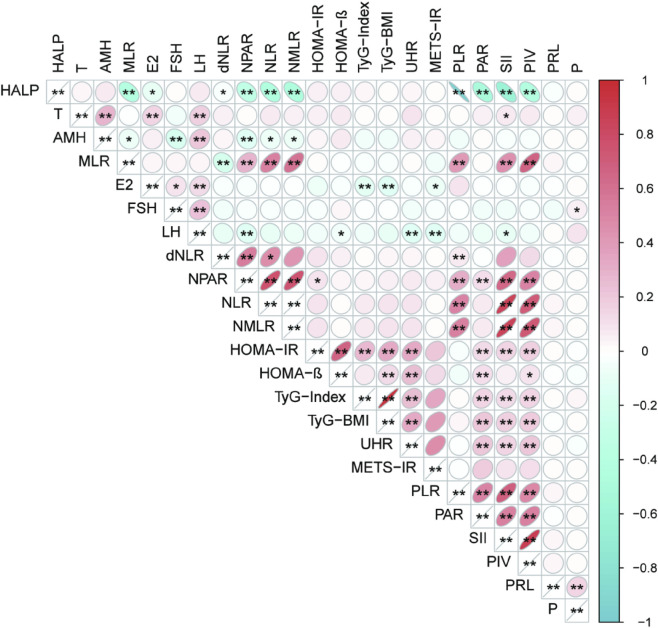
Correlation between basic sex hormones and new hematological parameters.

Spearman correlation analysis revealed that AMH level and MLR (*r* = 0.079, *P* = 0.035), NMLR (*r* = 0.080, *P* = 0.032), NLR (*r* = 0.076, *P* = 0.042), NPAR (*r* = 0.130, *P* < 0.001) showed a negative correlation; LH levels and NPAR (*r* = 0.128, *P* < 0.001), UHR (*r* = 0.127, *P* < 0.001), SII (*r* = 0.105, *P* = 0.005), HOMA - beta (*r* = 0.063, *P* = 0.092), the METS - IR (*r* = 0.142, *P* < 0.001) showed a negative correlation; The E2 level was negatively correlated with HALP (*r* = −0.087, *P* = 0.020), TyG Index (*r* = −0.108, *P* = 0.004), TyG-BMI (*r* = −0.128, *P* < 0.001), and METS-IR (*r* = −0.081, *P* = 0.030). The T level was positively correlated with SII (*r* = 0.075, *P* = 0.045), but FSH, PRL, and P were not correlated with inflammation and insulin resistance-related indicators. Notably, inflammatory markers (UHR, PAR, PIV) positively correlated with IR indices (HOMA-IR, HOMA-β, TyG Index, TyG-BMI). Additionally, SII was positively associated with HOMA-IR, TyG Index, and TyG-BMI, while NPAR correlated with HOMA-β (*P* < 0.05; Figure 1).

### 3.5 Logistic regression analysis of novel hematological parameters

Hematological parameters were stratified into quartiles (Q1-Q4) based on control group distributions. After adjusting for age and BMI (Model 1), Q4 levels of TyG Index and TyG-BMI were positively associated with clinical pregnancy failure (*OR*: 1.74, 95% *CI*: 1.14–2.66; and *OR*: 2.04, 95% *CI*: 1.30–3.21, respectively), compared to Q1. Notably, while the overall trend tests for NLR (*P*_trend_ = 0.138) and NPAR (*P*_trend_ = 0.149) did not reach statistical significance, subsequent subgroup analyses demonstrated clinically meaningful patterns. the likelihood of clinical pregnancy failure was 69% greater for participants in the NLR Q3 group (*OR*: 1.69, 95% *CI*: 1.09–2.62; *P* = 0.020), and those in the NPAR Q4 group exhibited a 1.60-fold increase compared with Q1 (95% *CI*: 1.04–2.45; *P* = 0.031), as detailed in Table 4.

**TABLE 4:** Logistic regression analysis of novel hematological parameters in model 1: Adjusted for age and BMI.

Variables	Q1	Q2 [OR (95% *CI*)]	Q3 [*OR* (95% *CI*)]	Q4 [*OR* (95% *CI*)]	*P*_*trend*_
HALP	Ref.	1.06 (0.69, 1.64)	1.36 (0.89, 2.08)	1.11 (0.72, 1.71)	0.507
NLR	Ref.	1.31 (0.84, 2.06)	**1.69 (1.09, 2.62)**	1.40 (0.90, 2.18)	0.138
PLR	Ref.	1.51 (0.98, 2.32)	1.23 (0.80, 1.90)	1.04 (0.66, 1.62)	0.203
SII	Ref.	1.33 (0.86, 2.07)	1.41 (0.91, 2.20)	1.24 (0.79, 1.93)	0.456
PAR	Ref.	1.44 (0.95, 2.20)	0.93 (0.60, 1.46)	0.96 (0.61, 1.49)	0.133
PIV	Ref.	1.07 (0.70, 1.65)	1.08 (0.70, 1.66)	1.01 (0.66, 1.57)	0.981
UHR	Ref.	1.13 (0.73, 1.76)	0.96 (0.61, 1.51)	1.50 (0.96, 2.33)	0.157
NPAR	Ref.	1.13 (0.72, 1.78)	1.27 (0.81, 1.99)	**1.60 (1.04, 2.45)**	0.149
dNLR	Ref.	1.19 (0.74, 1.92)	1.31 (0.86, 2.02)	1.09 (0.71, 1.65)	0.617
MLR	Ref.	1.11 (0.71, 1.75)	1.28 (0.80, 2.03)	1.15 (0.73, 1.81)	0.773
NMLR	Ref.	1.39 (0.89, 2.17)	1.52 (0.98, 2.36)	1.42 (0.91, 2.21)	0.274
METSIR	Ref.	0.72 (0.46, 1.13)	1.14 (0.75, 1.73)	1.09 (0.71, 1.67)	0.198
TyG index	Ref.	0.84 (0.53, 1.34)	1.14 (0.73, 1.77)	**1.74 (1.14, 2.66)**	**0.005**
TyG-BMI	Ref.	0.93 (0.58, 1.49)	1.36 (0.86, 2.14)	**2.04 (1.30, 3.21)**	**0.002**
HOMA-IR	Ref.	1.09 (0.70, 1.69)	1.06 (0.68, 1.66)	1.25 (0.80, 1.94)	0.789
HOMA-β	Ref.	1.01 (0.66, 1.55)	1.00 (0.65, 1.55)	1.05 (0.68, 1.63)	0.996

After full adjustment for covariates, the strong associations persisted. Women in Q4 of TyG Index and TyG-BMI showed 77% (*OR*:1.77, 95%*CI*:1.13–2.77) and 95% (*OR*:1.95, 95%*CI*:1.21–3.15) higher odds of clinical pregnancy failure versus Q1, respectively (Table 5). Furthermore, PAR emerged as a significant correlate (*P*_trend_ = 0.044). Although NLR showed no significant overall trend (*P*_trend_ = 0.267), subgroup analysis indicated that the odds of pregnancy failure were 1.59 times higher in Q3 (95% *CI*: 1.00–2.53, *P* = 0.042) when compared to Q1.

**TABLE 5:** Logistic regression analysis of novel hematological parameters in model 2: Fully adjusted model^a^.

Variables	Q1	Q2 [*OR* (95% *CI*)]	Q3 [*OR* (95% *CI*)]	Q4 [*OR* (95% *CI*)]	*P*_trend_
HALP	Ref.	1.20 (0.76, 1.90)	1.50 (0.96, 2.36)	1.33 (0.84, 2.10)	0.335
NLR	Ref.	1.33 (0.83, 2.12)	**1.59 (1.00, 2.53)**	1.25 (0.78, 2.01)	0.267
PLR	Ref.	1.44 (0.92, 2.26)	1.14 (0.72, 1.81)	0.91 (0.56, 1.45)	0.194
SII	Ref.	1.43 (0.90, 2.26)	1.37 (0.86, 2.19)	1.14 (0.71, 1.83)	0.395
PAR	Ref.	1.67 (1.07, 2.61)	0.97 (0.61, 1.56)	1.03 (0.64, 1.64)	**0.044**
PIV	Ref.	1.09 (0.69, 1.71)	1.10 (0.70, 1.73)	1.02 (0.64, 1.61)	0.967
UHR	Ref.	0.96 (0.60, 1.52)	0.91 (0.56, 1.46)	1.41 (0.88, 2.24)	0.205
NPAR	Ref.	1.13 (0.70, 1.81)	1.29 (0.81, 2.05)	1.40 (0.89, 2.21)	0.488
dNLR	Ref.	1.13 (0.71, 1.79)	1.52 (0.97, 2.37)	1.06 (0.67, 1.68)	0.246
MLR	Ref.	0.77 (0.49, 1.22)	1.19 (0.76, 1.86)	0.95 (0.61, 1.48)	0.328
NMLR	Ref.	1.39 (0.87, 2.21)	1.39 (0.88, 2.22)	1.27 (0.79, 2.02)	0.477
METSIR	Ref.	0.73 (0.45, 1.17)	1.10 (0.70, 1.71)	1.08 (0.69, 1.69)	0.319
TyG index	Ref.	0.94 (0.58, 1.51)	1.02 (0.64, 1.64)	**1.77(1.13, 2.77)**	**0.012**
TyG-BMI	Ref.	1.01 (0.61, 1.65)	1.33 (0.83, 2.15)	**1.95(1.21, 3.15)**	**0.014**
HOMA-IR	Ref.	1.29 (0.81, 2.04)	1.15 (0.72, 1.84)	1.47 (0.92, 2.36)	0.419
HOMA-β	Ref.	1.03 (0.66, 1.62)	1.04 (0.66, 1.64)	1.02 (0.64, 1.61)	0.998

^a^ Model 2 was adjusted for age, BMI, infertility duration, fertilization method, number of treatments, reproductive hormones (AMH, FSH, LH, PRL, E_2_, T, P), stage of embryos transfer, number of embryos transfer, and reproductive comorbidities (PCOS, DOR, endometriosis, genital tract inflammation). The bold values indicate P<0.05.

### 3.6 The nonlinear regression analysis between novel hematological parameters and clinical pregnancy outcomes

**FIGURE 2:**
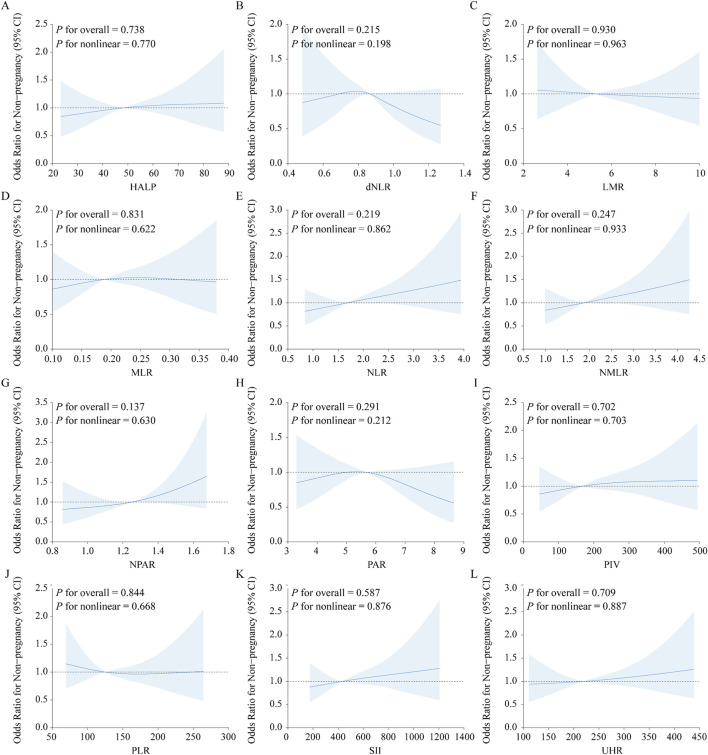
The RCS between inflammation-related indicators (**(A)** HALP, **(B)** dNLR, **(C)** LMR, **(D)** MLR, **(E)** NLR, **(F)** NMLR, **(G)** NPAR, **(H)** PAR, **(I)** PIV, **(J)** PLR, **(K)** SII, **(L)** UHR) and clinical pregnancy outcomes. The blue solid line represents the estimated Odds Ratio (OR), and the shaded area indicates the 95% confidence intervals. The model was adjusted for Age, BMI, infertility duration, fertilization method, treatment cycles, reproductive hormones (AMH, FSH, LH, PRL, E2, T, P), stage of embryos transfer, number of embryos transfer, and reproductive comorbidities (PCOS, DOR, endometriosis, inflammation of the reproductive system). The dotted line refers to the reference Odds Ratio (OR = 1.0).

**FIGURE 3:**
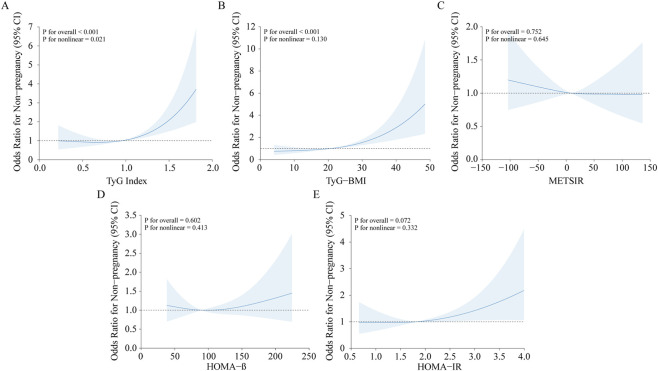
The RCS between insulin resistance-related indicators (**(A)** TyG Index, **(B)** TyG-BMI), **(C)** METSIR, **(D)** HOMA-β, **(E)** HOMA-IR) and clinical pregnancy outcomes. The blue solid line represents the estimated Odds Ratio (OR), and the shaded area indicates the 95% confidence intervals. The model was adjusted for Age, BMI, infertility duration, fertilization method, treatment cycles, reproductive hormones (AMH, FSH, LH, PRL, E2, T, P), stage of embryos transfer, number of embryos transfer, and reproductive comorbidities (PCOS, DOR, endometriosis, inflammation of the reproductive system). The dotted line refers to the reference Odds Ratio (OR = 1.0)

Restricted cubic spline (RCS) analysis was employed to assess potential nonlinear relationships between inflammatory/insulin resistance markers and FET outcomes (Figures 2, 3). After adjusting for all covariates, the TyG Index demonstrated a significant positive nonlinear association with clinical pregnancy failure (*P*_for overall_ <0.001, *P*_for nonlinear_ = 0.021), while TyG-BMI showed a positive linear relationship (*P*_for overall_ <0.001, *P*_for nonlinear_ = 0.130).

### 3.7 LASSO-logistic regression analysis of novel hematological parameters

Using clinical pregnancy failure as the dependent variable, LASSO regression with 10-fold cross-validation was performed for variable selection (Supplementary Table S3; Supplementary Figure S1; Supplementary Figure S2). The NPAR and TyG-BMI were identified. Subsequent multivariate logistic regression incorporating these markers with all covariates without BMI revealed age (*OR*:1.05, 95%*CI*:1.01–1.10), stage of embryos transfer (*OR*:0.49, 95%*CI*: 0.28–0.85), Q4 level of TyG-BMI (*OR*:2.11, 95%*CI*:1.34–3.31), as significant associations with pregnancy outcomes. And the results remained consistent after including BMI (Table 6).

**TABLE 6:** The results of the multivariate logistic regression analysis.

Variables	Model 1			Model 2		
	*OR* (95% *CI*)	*P*	*P*_trend_	*OR* (95% *CI*)	*P*	*P*_trend_
Age	1.05 (1.01,1.10)	0.020	​	**1.05 (1.01,1.10)**	**0.021**	​
BMI	-	-	-	1.16 (0.84,1.58)	0.367	​
Years of infertility	0.99 (0.92,1.08)	0.901	​	0.99 (0.92,1.08)	0.892	​
Fertilization method	0.71 (0.49,1.03)	0.072	​	0.70 (0.48,1.02)	0.066	​
Number of treatments	0.99 (0.85,1.15)	0.865	​	0.99 (0.85,1.15)	0.918	​
Stage of embryos transfer	**0.49 (0.28,0.85)**	**0.012**	​	**0.48 (0.27,0.84)**	**0.011**	​
Number of embryos transfer	1.19 (0.73,1.94)	0.475	​	1.19 (0.73,1.93)	0.491	​
Polycystic ovary syndrome	0.82 (0.54,1.23)	0.328	​	0.80 (0.53,1.20)	0.281	​
Diminished ovarian reserve	1.65 (0.92,2.96)	0.092	​	1.66 (0.93,2.98)	0.089	​
Endometriosis	0.98 (0.47,2.02)	0.953	​	0.98 (0.48,2.02)	0.955	​
Inflammation	0.82 (0.48,1.40)	0.471	​	0.82 (0.48,1.39)	0.465	​
AMH	0.98 (0.93,1.03)	0.508	​	0.98 (0.93,1.04)	0.52	​
FSH	1.07 (0.96,1.20)	0.209	​	1.07 (0.96,1.20)	0.223	​
LH	0.90 (0.81,1.00)	0.050	​	0.91 (0.82,1.01)	0.063	​
PRL	1.00 (0.97,1.02)	0.711	​	1.00 (0.97,1.02)	0.684	​
E_2_	1.00 (0.99,1.01)	0.858	​	1.00 (0.99,1.01)	0.923	​
T	1.32 (0.25,6.88)	0.739	​	1.27 (0.24,6.63)	0.779	​
P	0.58 (0.18,1.88)	0.362	​	0.62 (0.19,2.05)	0.434	​
NPAR	​	​	0.459	​	​	0.490
Q1	ref	​	​	​	​	​
Q2	1.14 (0.71, 1.84)	0.578	​	1.13 (0.70,1.82)	0.608	​
Q3	1.28 (0.80,2.04)	0.310	​	1.27 (0.80,2.04)	0.312	​
Q4	1.43 (0.91,2.27)	0.124	​	1.41 (0.89,2.24)	0.142	​
TyG-BMI	​	​	**0.002**	​	​	**0.014**
Q1	ref	​	​	​	​	​
Q2	1.02 (0.62, 1.68)	0.930	​	1.01 (0.62,1.66)	0.963	​
Q3	1.38 (0.86,2.21)	0.184	​	1.34 (0.83,2.15)	0.235	​
Q4	**2.11 (1.34,3.31)**	**0.001**	​	**1.96 (1.21,3.16)**	**0.006**	​

Model 1 was adjusted for age, infertility duration, fertilization method, number of treatments, reproductive hormones (AMH, FSH, LH, PRL, E_2_, T, P), stage and number of embryos transfer, and reproductive comorbidities (PCOS, DOR, endometriosis, genital tract inflammation). Model 2 was adjusted for age, BMI, infertility duration, fertilization method, number of treatments, reproductive hormones (AMH, FSH, LH, PRL, E_2_, T, P), stage of embryos transfer, number of embryos transfer, and reproductive comorbidities (PCOS, DOR, endometriosis, genital tract inflammation). The bold values indicate P<0.05.

### 3.8 Weighted quantile sum regression analysis of novel hematological parameters

#### 3.8.1 Weighted quantile sum regression analysis of inflammation-related indicators

**FIGURE 4:**
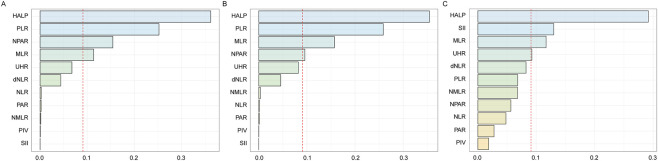
WQS Weight Bar Chart of Inflammation-Related Indicators **(A)** Unadjusted; **(B)** Adjusted for age, BMI, duration of infertility, fertilization methods, and number of treatments; **(C)** Adjusted for age, BMI, infertility duration, fertilization method, number of treatments, reproductive hormones (AMH, FSH, LH, PRL, E2, T, P), stage of embryos transfer, number of embryos transfer, and reproductive comorbidities (PCOS, DOR, endometriosis, genital tract inflammation). The bar chart displays the estimated relative weight of each individual indicator contributing to the WQS index. The red dashed line denotes the theoretical threshold for equal contribution (1/c = 0.09, where c is the total number of components included in the model). Variables with estimated weights exceeding this prespecified threshold were considered the predominant contributors to the overall mixture effect.

The continuous variables of the inflammatory markers were transformed into quartiles to construct the WQS index. In unadjusted WQS analysis, each quartile increase in the WQS index of inflammatory markers was associated with a 2.49-fold elevated risk of clinical pregnancy failure (95%*CI*: 1.28–4.84, *P* = 0.007), with HALP demonstrating the highest weighted component (0.36, Figure 4A). However, after comprehensive adjustment for age, BMI, infertility duration, fertilization method, number of treatments, stage, and number of embryos, and reproductive hormones, this association was attenuated and no longer statistically significant (Figure 4C).

#### 3.8.2 Weighted quantile sum regression analysis of insulin resistance-related indicators

**FIGURE 5:**
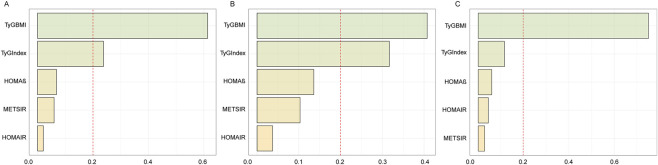
WQS Weight Bar Chart of Insulin Resistance-Related Indicators. **(A)** Unadjusted; **(B)** Adjusted for age, BMI, infertility duration, fertilization methods, and number of treatments; **(C)** Adjusted for age, BMI, infertility duration, fertilization method, number of treatments, reproductive hormones (AMH, FSH, LH, PRL, E2, T, P), stage of embryos transfer, number of embryos transfer, and reproductive comorbidities (PCOS, DOR, endometriosis, genital tract inflammation). The bar chart displays the estimated relative weight of each individual indicator contributing to the WQS index. The red dashed line denotes the theoretical threshold for equal contribution (1/c =0.2, where c is the total number of components included in the model). Variables with estimated weights exceeding this prespecified threshold were considered the predominant contributors to the overall mixture effect.

In the unadjusted WQS model, each quartile increase in the WQS index was associated with a 1.62-fold higher risk of clinical pregnancy failure (95%*CI*:1.32–1.99, *P* < 0.001), with TyG-BMI demonstrating the highest weighted contribution (0.61) followed by TyG Index (0.24; Figure 5A). After adjustment for age, BMI, infertility duration, fertilization method, and number of treatments, the WQS index remained significantly associated with pregnancy failure (*OR*:1.63 per quartile, 95%*CI*:1.28–2.07, *P* < 0.001), where age showed an independent positive association (*P* = 0.025). In this model, TyG-BMI maintained the dominant weight (0.41) with TyG Index secondary (0.32; Figure 5B). Further adjustment for more covariates attenuated but preserved the WQS association (*OR*: 1.31, 95%*CI*:1.04–1.65, *P* < 0.001).

### 3.9 Incremental predictive value and clinical magnitude of TyG-BMI for pregnancy outcomes in frozen embryo transfer

To further evaluate the incremental predictive value of metabolic indices, the TyG Index and TyG-BMI were sequentially incorporated into the baseline clinical models (Supplementary Table S4). Adding the TyG Index to baseline Model 1 (which included BMI) showed incremental predictive value in this dataset (NRI = 0.262, *P* < 0.001; IDI = 0.012, *P* = 0.004), despite a non-significant increase in discrimination (ΔAUC = 0.014, *P* = 0.107). Conversely, introducing TyG-BMI into baseline Model 2 (which excluded BMI to prevent over-adjustment) yielded significant enhancements in both discrimination (ΔAUC = 0.023, *P* = 0.047) and reclassification metrics (NRI = 0.264, *P* < 0.001; IDI = 0.020, *P* < 0.001). Furthermore, sensitivity analyses corroborated that the predictive efficacy of TyG-BMI is independent of BMI and biochemical pregnancy (Supplementary Tables S5, 6). After excluding baseline BMI, the highest TyG-BMI quartile (Q4) remained consistently associated with an increased risk of adverse pregnancy outcomes across all configurations (*OR*: 2.11–2.20, *P*_trend_ < 0.01 for all). Similarly, further exclusion of biochemical pregnancy also resulted in the same outcome. To translate these findings into clinical magnitude, we calculated the marginal predicted probabilities across TyG-BMI quartiles, holding all other covariates in the fully adjusted model at their means. The predicted probability of clinical pregnancy notably decreased from 63.1% in the lowest quartile (Q1) to 46.6% in the highest quartile (Q4), yielding a substantial absolute risk reduction of 16.5% (Supplementary Figure S4). Collectively, these findings highlight the robustness of TyG-BMI as an independent predictor of adverse outcomes in FET cycles.

## 4 Discussion

### 4.1 Age, ovarian reserve, and immunometabolic dysregulation

Multiple studies have established maternal age as an independent risk factor for ART success. A large prospective cohort study of 2,193 women undergoing donor insemination for male-factor infertility demonstrated age-stratified cumulative pregnancy rates of 74% (<31 years), 62% (31–35 years), and 54% (>35 years) after 12 cycles, with this decline persisting after adjustment for paternal factors (Schwartz and Mayaux, 1982). Similarly, a retrospective analysis of 20,806 oocyte retrieval cycles revealed that advanced maternal age correlated with diminished AMH levels, poorer oocyte/embryo quality, and elevated embryonic aneuploidy risk, collectively reducing live birth rates (Gao et al., 2024). These findings align with multinational large-scale studies and our data, supporting age as a significant predictor of compromised FET outcomes (Goldman et al., 2014; Hornstein, 2016; Wennberg et al., 2016).

In our cohort, the non-pregnancy group exhibited significantly higher age, BMI, leukocyte parameters, and inflammatory/insulin resistance markers. Age-related physiological decline manifests through two synergistic pathways. First, reduced lipoprotein lipase activity promotes adiposity, while adipose tissue macrophages secrete MCP-1 to activate NF-κB/JNK pathways. This elevates proinflammatory cytokines (TNF-α, IL-6) that may impair insulin signaling via suppressed receptor phosphorylation and GLUT4 translocation (Lu et al., 2019; Li et al., 2020). Second, degenerative changes in glucoregulatory enzymes exacerbate glycemic instability, creating a vicious cycle of hyperinsulinemia-driven lipogenesis and inflammation. These mechanisms parallel our Spearman correlations, which showed consistent positive associations between inflammatory indices (e.g., UHR, PIV, SII) and insulin resistance markers (e.g., HOMA-IR, TyG Index). While ovarian aging remains the primary explanation for age-related fertility decline, our findings suggest that immunometabolic dysregulation may potentiate this process.

### 4.2 Insulin resistance and metabolic markers

In our study, the TyG Index and TyG-BMI were significantly associated with the clinical pregnancy outcomes, whereas HOMA-IR was not. This discrepancy likely arises because the triglyceride-driven TyG Index is a more sensitive surrogate for peripheral insulin resistance and systemic lipotoxicity compared to the insulin-dependent, hepatic-focused HOMA-IR. Our multivariable logistic regression analyses demonstrated robust positive associations between insulin resistance markers (TyG Index and TyG-BMI) and FET failure risk across adjusted models. Notably, RCS analysis revealed distinct dose-response relationships - a nonlinear positive association for TyG Index versus a linear relationship for TyG-BMI. The clinical relevance of TyG-BMI was further corroborated by its selection in LASSO regression for variable importance.

Insulin resistance adversely affects fertility in reproductive-aged women and has been associated with compromised clinical outcomes in ART. Takikawa et al. demonstrated lower pregnancy rates in cycles with elevated follicular fluid insulin, suggesting that ovarian IR may negatively impact IVF-ET success (Takikawa et al., 2010). Prospective cohort studies confirm metabolic coupling between serum and follicular microenvironments, where altered metabolite profiles correlate with oocyte quality (Valckx et al., 2012). Animal models reveal that IR-induced metabolic alterations are associated with developmental delays across embryonic stages (Minge et al., 2008). Clinically, while IR shows minimal impact on oocyte maturation in PCOS patients, it has been linked to reduced implantation rates, pointing toward endometrial dysfunction as a key mechanism (Chang et al., 2013).

The pathophysiological relationship between IR, compensatory hyperinsulinemia, and sex hormone regulation remains incompletely understood. *In vitro* studies demonstrate insulin’s dual role in stimulating granulosa cell estradiol production while potentiating FSH-induced steroidogenesis, potentially contributing to dyssynchronous follicular development (Willis et al., 1996; Mason et al., 1994; Franks et al., 1996). Furthermore, IR may modulate androgen levels via hepatic sex hormone-binding globulin suppression, which could exacerbate ovulation dysfunction (Diamanti-Kandarakis and Dunaif, 1996; Moghetti et al., 1996). Compared to conventional measures (QUICKI, FG-IR), the triglyceride-glucose indices (TyG Index and TyG-BMI) offer a simple, cost-effective alternative for IR screening (Zheng et al., 2016; Sánchez-García et al., 2020; Simental-Mendía et al., 2020; Zheng et al., 2022; Mazidi et al., 2018). From a clinical perspective, these indices offer easily accessible screening tools for metabolic dysfunction in reproductive medicine. Evaluating these indices before frozen-thawed embryo transfer may help identify patients at higher risk of implantation failure. Consequently, this allows for the timely implementation of pre-conceptional interventions, such as tailored lifestyle modifications or pharmacological insulin sensitization, to optimize the maternal metabolic microenvironment and potentially improve clinical pregnancy outcomes.

From an epidemiological perspective, identifying the precise causal structure between metabolic indices and IVF outcomes is challenging. In our assumed causal framework (based on Directed Acyclic Graph principles), conditions such as PCOS and BMI are intimately correlated with our primary exposures (TyG Index) and may partially lie on the causal pathway. The robust associations observed in our unadjusted and partially adjusted models reflect the total effect of this metabolic-inflammatory environment. Crucially, the persistence of these associations even after adjusting for PCOS, DOR, and endometriosis suggests that insulin resistance exerts a direct detrimental effect on the early window of implantation and endometrial receptivity, independent of the original cause of infertility (Ding et al., 2025). Additionally, sensitivity analyses confirming the predictive value of TyG-BMI without the over-adjustment of baseline BMI further validate its independent utility. Importantly, the specific context of our cohort, utilizing artificial hormone replacement therapy (HRT) for FET, further reinforces this mechanism. In an HRT-FET model, endogenous ovarian function is pharmacologically suppressed (Pan et al., 2020), and embryo quality is predetermined before warming. Therefore, the detrimental effects of elevated TyG Index observed in this study are highly unlikely to be driven by the immediate ovarian microenvironment. Instead, they strongly suggest that systemic metabolic dysfunction primarily impairs endometrial receptivity and disrupts the local immune tolerance necessary for early trophoblast invasion.

### 4.3 Systemic inflammatory indices and mixed exposures

Beyond metabolic parameters, multivariable logistic regression identified PAR as an independent risk factor for FET failure, with Q2 levels conferring significantly elevated risk versus Q1. Furthermore, LASSO regression selected NPAR as a key predictor. As noted in our results, while the unadjusted WQS analysis indicated a 2.49-fold increased FET failure risk per quartile of the inflammatory mixture (with the HALP score as the predominant driver), this overarching association was attenuated after comprehensive adjustment for clinical covariates. Nevertheless, these hematological indices (NLR, NPAR, PAR, PIV, HALP), derived from routine complete blood counts, offer universally accessible measures of systemic inflammation. While extensively validated in oncological and autoimmune diseases (Liu et al., 2024), their role in ART remains underexplored. The underlying mechanisms may involve chronic low-grade inflammation impairing embryogenesis or endometrial receptivity (Rodriguez et al., 2026), a hypothesis that warrants further investigation in larger cohorts. Specifically, indices such as NPAR, PAR, and NLR are driven by peripheral neutrophils and systemic protein levels. Neutrophils are primary sources of reactive oxygen species (ROS) and proinflammatory cytokines (such as TNF-α and IL-6), which can penetrate the reproductive tract and disrupt the endometrial immune balance (Wang et al., 2026). Elevated systemic neutrophils (reflected in high NLR or NPAR) coupled with diminished prealbumin or lymphocytes (reflected in low HALP scores) indicate a shift toward a hyper-inflammatory and nutritionally depleted systemic state. This circulating cytokine milieu directly bathes the endometrial vasculature, potentially upregulating pro-apoptotic pathways within the decidua and creating a hostile microenvironment for embryo implantation.

### 4.4 Limitations

Our study has several limitations that should be explicitly acknowledged. First, the retrospective, single-center design inherently carries the risk of selection bias and residual confounding. While our models rigorously adjusted for primary clinical and embryological covariates (including DAG-guided covariate selection and WQS regression), unmeasured lifestyle factors, such as specific dietary habits, physical activity levels, psychological stress, and concurrent medication use (e.g., metformin, statins, or anti-inflammatory drugs), could not be entirely accounted for. Second, our primary outcome was limited to clinical pregnancy. While this serves as a critical indicator of successful initial embryo implantation and endometrial receptivity, it does not perfectly surrogate the ultimate goal of ART—live birth. Miscarriages or early pregnancy losses occurring after the first ultrasound confirmation were not captured in this study. Third, our hematological and metabolic indices were derived from a single fasting blood sample collected before embryo transfer. This single temporal snapshot may be susceptible to temporal misclassification and might not fully reflect the dynamic physiological fluctuations occurring during the critical peri-implantation window. Fourth, our cohort consisted entirely of an Asian population undergoing artificial HRT-FET; thus, the generalizability of our findings to other ethnicities or alternative endometrial preparation protocols remains to be determined. Finally, without direct molecular assessments of endometrial biopsies, follicular fluid cytokine assays, or detailed embryonic morphokinetics, our inferences regarding the underlying pathophysiological mechanisms linking systemic hematological parameters to local reproductive pathophysiology remain speculative. Future prospective, longitudinal cohorts with complete follow-up to live birth and neonatal outcomes are urgently warranted to validate our findings and determine whether therapeutic interventions modulating these inflammatory and metabolic pathways could definitively improve ART outcomes.

## 5 Conclusion

Elevated TyG Index and TyG-BMI are significantly associated with adverse clinical pregnancy outcomes following FET. These findings serve as a hypothesis-generating foundation, suggesting that these metabolic indices might act as potential correlative markers. However, they should not yet be utilized as standalone clinical decision tools in practice. Future large-scale, prospective validation studies are required to confirm their clinical utility.

## Data Availability

The original contributions presented in the study are included in the article/Supplementary Material, further inquiries can be directed to the corresponding author.
