# Supplementary material for: Correlation between novel hematological parameters and assisted pregnancy outcome in frozen-thawed embryo transfer patients
Source: Front Cell Dev Biol. 2026 Jun 2;14:1781939. doi: 10.3389/fcell.2026.1781939 (PMC13268793; doi:10.3389/fcell.2026.1781939)
Supplement: Supplementary file 1 [file DataSheet1.docx]

**Supplementary Table S1. Novel hematological parameter calculation formula**

| Index | Formula |
| --- | --- |
| HALP | Hb (g/L) × Albumin (g/L) × ALC (×10⁹/L) / PLT (×10⁹/L) |
| NLR | ANC (×10⁹/L) / ALC (×10⁹/L) |
| LMR | ALC (×10⁹/L) / AMC (×10⁹/L) |
| PLR | PLT (×10⁹/L) / ALC (×10⁹/L) |
| SII | PLT(×10⁹/L) × ANC (×10⁹/L) / ALC (×10⁹/L) |
| PAR | PLT (×10⁹/L) / Albumin (g/L) |
| PIV | (ANC × AMC × PLT) (×10⁹/L) / ALC (×10⁹/L) |
| UHR | Uric acid (μmol/L) / HDL-C (mmol/L) |
| NPAR | NE%/ Albumin (g/L) |
| dNLR | ANC (×10⁹/L) / (WBC (×10⁹/L) – ALC (×10⁹/L)) |
| MLR | AMC (×10⁹/L) / ALC (×10⁹/L) |
| NMLR | (AMC + ANC) (×10⁹/L) / ALC (×10⁹/L) |
| TyG Index | ln[Fasting triglycerides (mg/dL) × Fasting glucose (mg/dL) / 2] |
| TyG-BMI | TyG Index × BMI (kg/m²) |
| HOMA-IR | [Fasting glucose (mmol/L) × Fasting insulin (μU/mL)] / 22.5 |
| HOMA-β | 20 × Fasting insulin (μU/mL) / (Fasting glucose (mmol/L) – 3.5) |
| METS-IR | [ln(2 × Fasting glucose (mmol/L) + ln(Triglycerides (mg/dL) × BMI)] / ln(HDL-C (mmol/L)) |

| **Supplementary Table S2. Embryo transfer details and clinical outcomes by pregnancy status** | | | | |
| --- | --- | --- | --- | --- |
|  | Clinical pregnancy group(N=404) | Non-pregnancy group(N=313) | Effect size (95% *CI*) | *P* |
| **Embryo transfer characteristics** |  |  |  |  |
| Number of embryos transferred, n(%) |  |  | 19.1% (10.2% to 27.9%) | **<0.001** |
| Single (1) | 332 (82.2) | 213 (68.1%) |  |  |
| Double (2) | 72 (17.8) | 100 (31.9%) |  |  |
| Stage of embryos transferred, n(%) |  |  | -30.1% (-39.2% to -20.9%) | **<0.001** |
| Cleavage stage (D3) | 45 (11.1) | 95 (30.4) |  |  |
| Blastocyst stage (D5/D6) | 359 (88.9) | 218 (69.6) |  |  |
| **Downstream clinical outcomes** |  |  |  |  |
| Biochemical pregnancy, n(%) * |  |  | - | - |
| yes | 0 (0.0) | 41 (13.1) |  |  |
| no | 0 (0.0) | 272 (86.9) |  |  |
| Ectopic pregnancy, n(%) † |  |  | - | - |
| yes | 1 (0.2) | 0 (0.0) |  |  |
| no | 403 (99.8) | 0 (0.0) |  |  |
| Miscarriage, n(%) ‡ |  |  | - | - |
| yes | 67 (16.6) | 0 (0.0) |  |  |
| no | 337 (83.4) | 0 (0.0) |  |  |
| Live birth, n(%) § |  |  |  |  |
| yes | 328 81.2) | 0 (0.0) |  |  |
| no | 76 (18.8) | 0 (0.0) |  |  |

Note: Data are presented as n (%). The *P*-value was calculated using the Chi-square test. *: Some biochemical pregnancies may not progress to clinical pregnancy, hence their presence in the Non-pregnancy Group. †, ‡, §: Downstream outcomes (ectopic pregnancy, miscarriage, and live birth) are logically strictly confined to patients who achieved a biochemical/clinical pregnancy. Thus, *P*-values between the clinical and non-clinical pregnancy groups for these specific outcomes are not applicable (-).

| **Supplementary Table S3. Full list of candidate variables entered into the LASSO regression model.** | |
| --- | --- |
| Variable Name | Abbreviation |
| neutrophil proportion to albumin ratio | HALP |
| neutrophil-to-lymphocyte ratio | NLR |
| lymphocyte-to-monocyte ratio | LMR |
| platelet-to-lymphocyte ratio | PLR |
| systemic immune-inflammation index | SII |
| platelet-to-albumin ratio | PAR |
| pan-immune inflammation value | PIV |
| uric acid to high-density lipoprotein cholesterol ratio | UHR |
| neutrophil proportion to albumin ratio | NPAR |
| derived neutrophil-to-lymphocyte ratio | dNLR |
| monocyte-to-lymphocyte ratio | MLR |
| neutrophil-monocyte-to-lymphocyte ratio | NMLR |
| metabolic score for insulin resistance | METS-IR |
| triglyceride glucose index | TyG Index |
| triglyceride glucose-body mass index | TyG-BMI |
| homeostasis model assessment of insulin resistance | HOMA-IR |
| homeostasis model assessment of beta cell Function | HOMA-β |

| **Supplementary Table S4.** **Incremental predictive value of adding TyG-BMI to the baseline clinical model for adverse pregnancy outcomes** | | | | | | |
| --- | --- | --- | --- | --- | --- | --- |
| Model | AUC (95% *CI*) | P for ΔAUC | NRI (95% *CI*) | P for NRI | IDI (95% *CI*) | P for IDI |
| Baseline Model 1 | 0.666 (0.626–0.705) | Ref. | Ref. | **-** | Ref. | **-** |
| Baseline Model 1  (+ TyG Index) | 0.680 (0.640–0.719) | 0.107 | 0.262 (0.125–0.399) | **< 0.001** | 0.012 (0.004–0.020) | **0.004** |
| Baseline Model2 | 0.653  (0.613–0.694) | Ref. | Ref. | **-** | Ref. | **-** |
| Enhanced Model2 (+ TyG-BMI) | 0.677  (0.637–0.716) | 0.047 | 0.264  (0.118–0.410) | **< 0.001** | 0.020  (0.010–0.031) | **< 0.001** |

Note: Baseline Model 1 included age, BMI, infertility duration, fertilization method, number of treatments, reproductive hormones (AMH, FSH, LH, PRL, E2, T, P), stage of embryos transfer, number of embryos transfer, and reproductive comorbidities (PCOS, DOR, endometriosis, genital tract inflammation). Baseline Model2 included age, infertility duration, fertilization method, number of treatments, reproductive hormones (AMH, FSH, LH, PRL, E2, T, P), stage of embryos transfer, number of embryos transfer, and reproductive comorbidities (PCOS, DOR, endometriosis, genital tract inflammation).

| **Supplementary Table S5. Sensitivity analysis excluding baseline BMI** | | | | | |
| --- | --- | --- | --- | --- | --- |
|  | Q1 | Q2 [*OR*(95% *CI*)] | Q3 [*OR*(95% *CI*)] | Q4 [*OR*(95% *CI*)] | *Ptrend* |
| TyG-BMI^a^ | Ref. | 0.95 (0.59, 1.53) | 1.42 (0.91, 2.22) | **2.20 (1.44, 3.38)** | **<0.001** |
| TyG-BMI^b^ | Ref. | 1.02(0.62, 1.67) | 1.38(0.86, 2.21) | **2.11(1.35, 3.32)** | **0.0018** |
| TyG-BMI^c^ | Ref. | 1.02(0.62, 1.68) | 1.38(0.86, 2.21) | **2.11(1.34, 3.31)** | **0.0021** |

^a^ Logistic regression analysis of TyG BMI adjusted for age.

^b^Logistic regression analysis of TyG BMI adjusted for age, infertility duration, fertilization method, treatment cycles, reproductive hormones (AMH, FSH, LH, PRL, E_2_, T, P), stage of embryos transfer, number of embryos transfer, and reproductive comorbidities (PCOS, DOR, endometriosis, genital tract inflammation).

^c^ multivariate logistic regression incorporating TyG-BMI and NPAR with age, infertility duration, fertilization method, treatment cycles, reproductive hormones (AMH, FSH, LH, PRL, E_2_, T, P), stage of embryos transfer, number of embryos transfer, and reproductive comorbidities (PCOS, DOR, endometriosis, genital tract inflammation).

| **Supplementary Table S6. Sensitivity analysis excluding the patient with biochemical pregnancy** | | | | | |
| --- | --- | --- | --- | --- | --- |
|  | Q1 | Q2 [*OR*(95% *CI*)] | Q3 [*OR*(95% *CI*)] | Q4 [*OR*(95% *CI*)] | *Ptrend* |
| TyG-BMI^a^ | Ref. | 0.93 (0.57, 1.51) | 1.27 (0.80, 2.04) | 2.08 (1.33, 3.24) | **<0.001** |
| TyG-BMI^b^ | Ref. | 0.99 (0.59, 1.66) | 1.18 (0.72, 1.96) | 1.76 (1.07, 2.91) | **0.0130** |
| TyG-BMI^c^ | Ref. | 0.99 (0.59, 1.67) | 1.19 (0.72, 1.97) | 1.77 (1.07, 2.92) | **0.0130** |

^a^ Logistic regression analysis of TyG BMI adjusted for age.

^b^Logistic regression analysis of TyG BMI adjusted for age, infertility duration, fertilization method, treatment cycles, reproductive hormones (AMH, FSH, LH, PRL, E_2_, T, P), stage of embryos transfer, number of embryos transfer, and reproductive comorbidities (PCOS, DOR, endometriosis, genital tract inflammation).

^c^ multivariate logistic regression incorporating TyG-BMI and NPAR with age, infertility duration, fertilization method, treatment cycles, reproductive hormones (AMH, FSH, LH, PRL, E_2_, T, P), stage of embryos transfer, number of embryos transfer, and reproductive comorbidities (PCOS, DOR, endometriosis, genital tract inflammation).


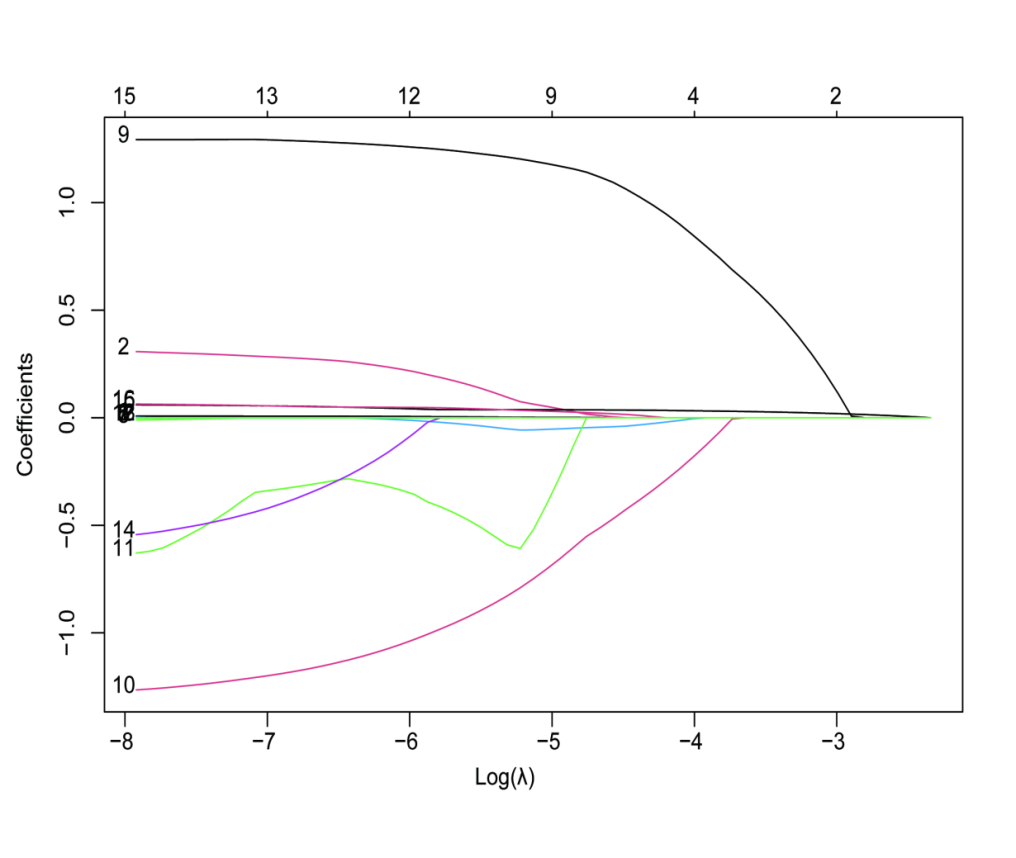


Supplementary Figure 1. Log (λ) and LASSO regression coefficient


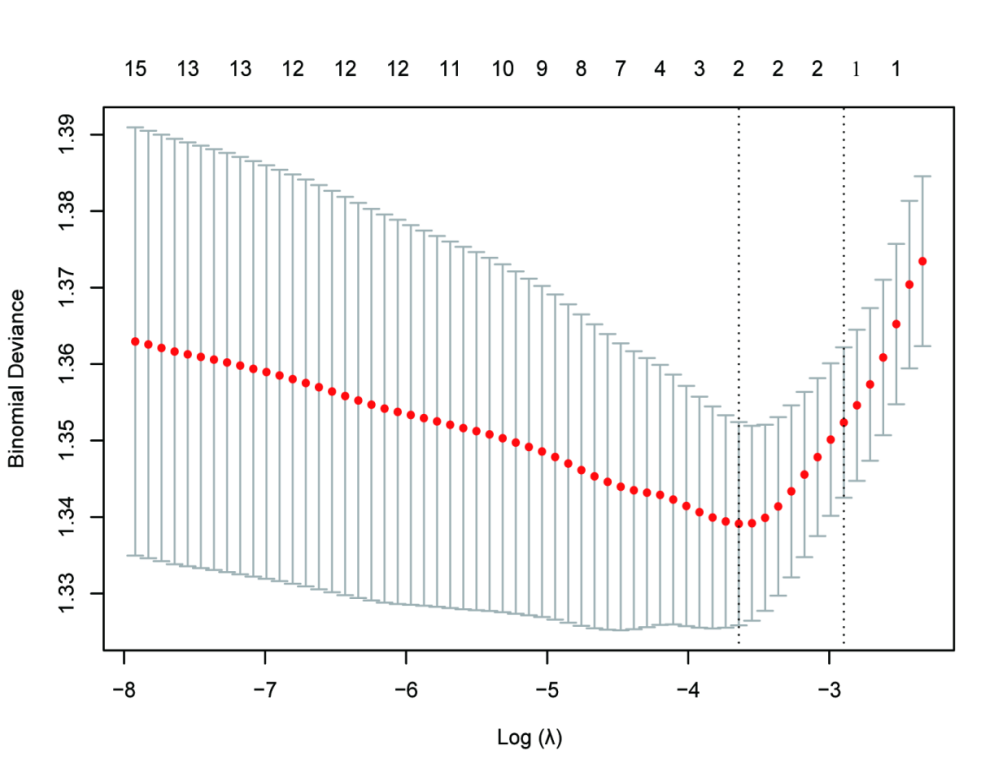


Supplementary Figure 2. Log(λ) and model error


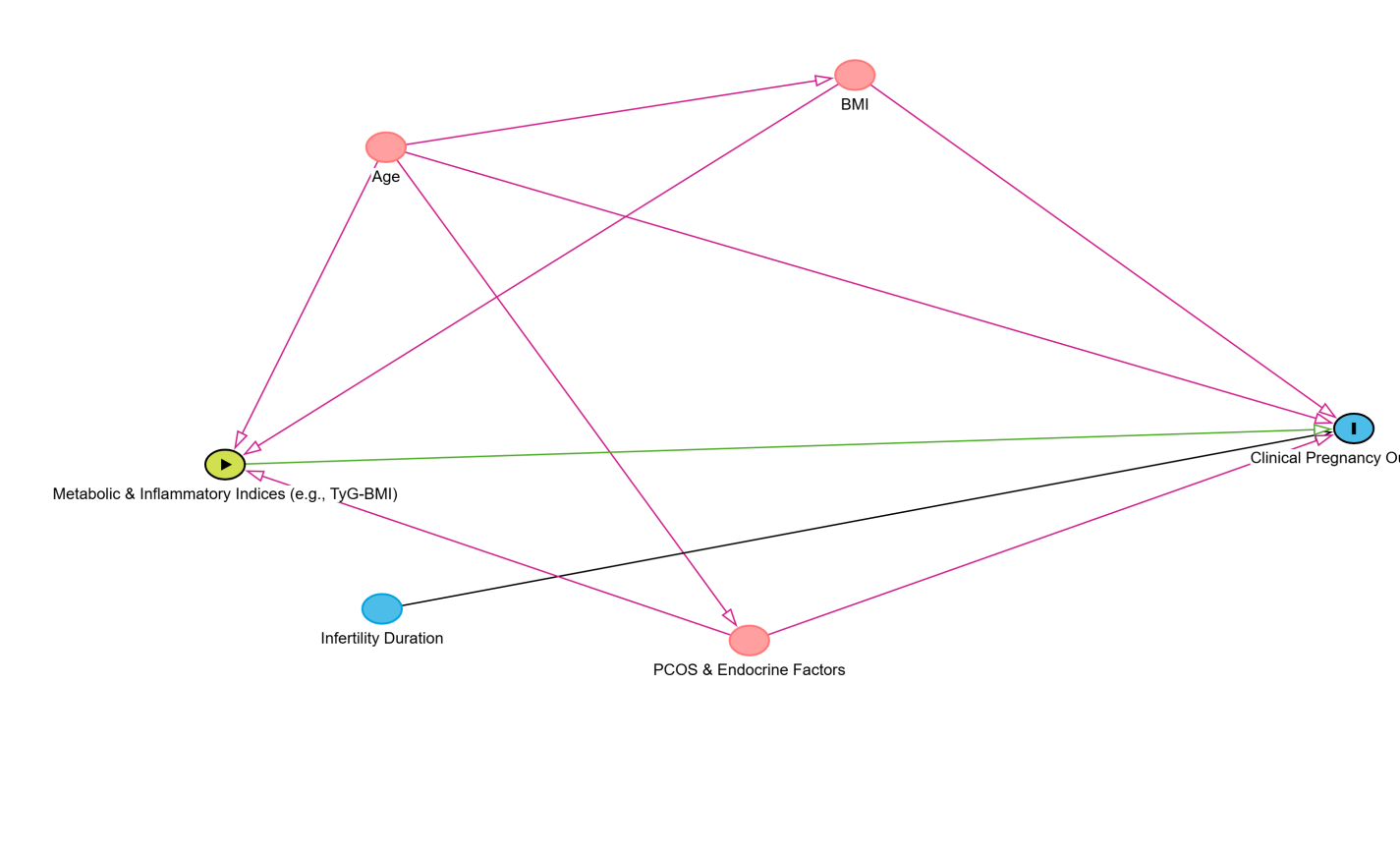


Supplementary Figure S3. Directed Acyclic Graph (DAG) illustrating the assumed causal framework. The DAG visualizes the relationship between the primary exposures (metabolic and inflammatory indices, e.g., TyG-BMI), the clinical pregnancy outcomes, and potential confounders or mediators. Maternal age and infertility duration are treated as fundamental ancestors. Baseline BMI and reproductive pathologies (e.g., PCOS) are depicted as complex nodes that potentially affect both the systemic metabolic state and endometrial receptivity. This structured framework justifies our sequential modeling approach to isolate direct vs. total effects and mitigate the risk of over-adjustment.


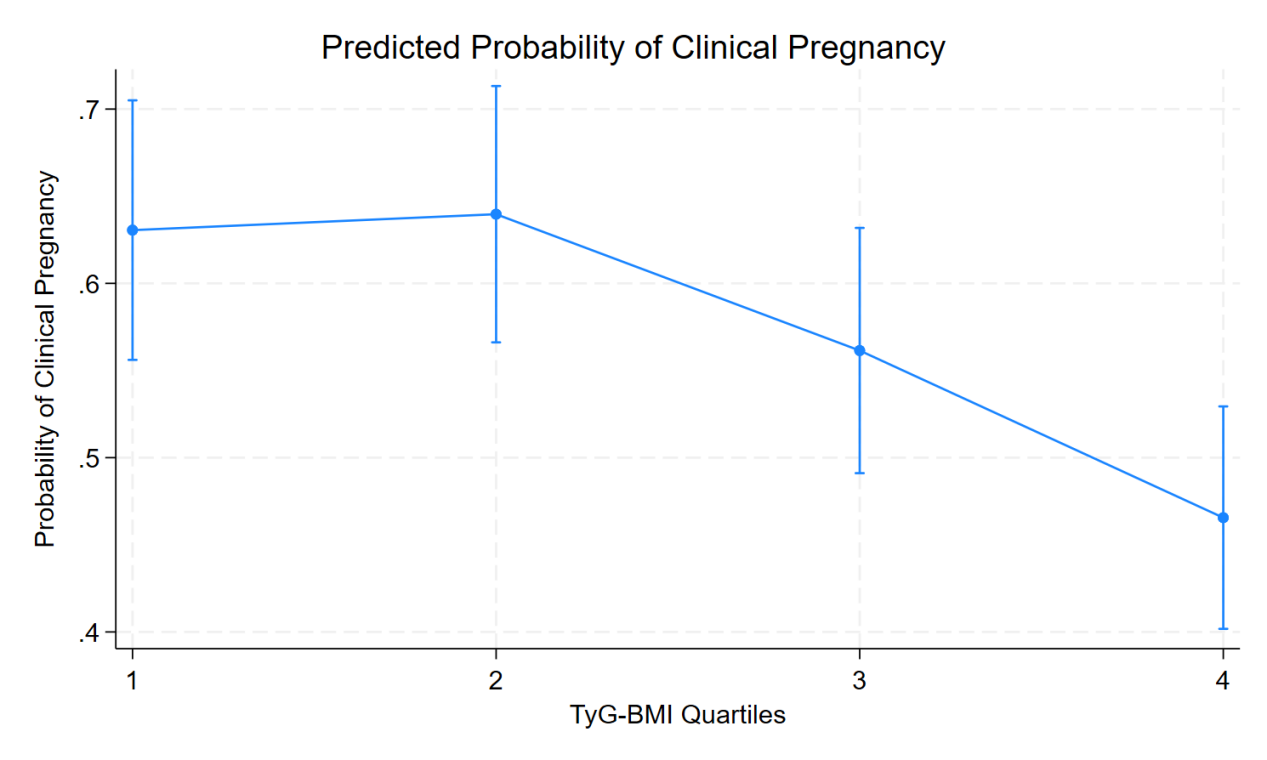


Supplementary Figure S4. The predicted probability of clinical pregnancy of TyG-BMI quartiles adjusted for age, BMI, infertility duration, fertilization method, number of treatments, reproductive hormones (AMH, FSH, LH, PRL, E2, T, P), stage of embryos transfer, number of embryos transfer, and reproductive comorbidities (PCOS, DOR, endometriosis, genital tract inflammation).
